# Tackle the Meckel: laparoscopic diverticulectomy for a retained ingested foreign body—a case report

**DOI:** 10.3389/fmed.2025.1650643

**Published:** 2025-10-01

**Authors:** Vania Myralda Giamour Marbun, Azzahra Fadhilah, Kezia Nathania Limbong Allo, Ahmad Aulia Ghufron, Mohammad Febriadi Ismet, Faris Nagib, Karl Heinz Leonhardt Rowika, Yarman Mazni

**Affiliations:** ^1^Department of Surgery, Digestive Surgery Division, Faculty of Medicine Universitas Indonesia, Cipto Mangunkusumo Hospital, Jakarta, Indonesia; ^2^Department of Surgery, Digestive Surgery Division, Cipto Mangunkusumo Hospital, Jakarta, Indonesia

**Keywords:** sharp object ingestion, retained foreign body, Meckel’s diverticulum, case report, adolescent

## Abstract

We report a 16-year-old female who ingested a headscarf pin that remained in the gastrointestinal tract for three weeks. Imaging suggested its presence in the distal ileum, and laparoscopy with fluoroscopy identified the pin lodged in a Meckel’s diverticulum. A hybrid laparoscopic-assisted diverticulectomy was performed, with uneventful recovery. This case emphasizes considering Meckel’s diverticulum in prolonged foreign body retention, as it may be missed on standard advanced imaging, and the value of minimally invasive surgery for definitive management.

## Introduction

Foreign body ingestion is a common gastrointestinal emergency, especially among pediatric patients, with up to 75% of cases occurring in children under four years of age and a similar incidence rate across genders (1, 2). A significant (approximately 80%) of ingested foreign bodies pass spontaneously through the gastrointestinal tract without intervention (3). However, ingestion of sharp, pointed, or large objects markedly elevates the risk of complications, including perforation, aspiration, and gastrointestinal bleeding. Early presentation to the emergency department for endoscopic retrieval is crucial in reducing the likelihood of adverse outcomes. When endoscopy fails to visualise or retrieve the foreign body, serial radiographic monitoring becomes essential to assess for spontaneous passage. In cases where passage does not occur naturally, surgical removal may be warranted (3). This case report has been reported in line with the SCARE checklist (4).

## Case presentation

**Figure 1:**
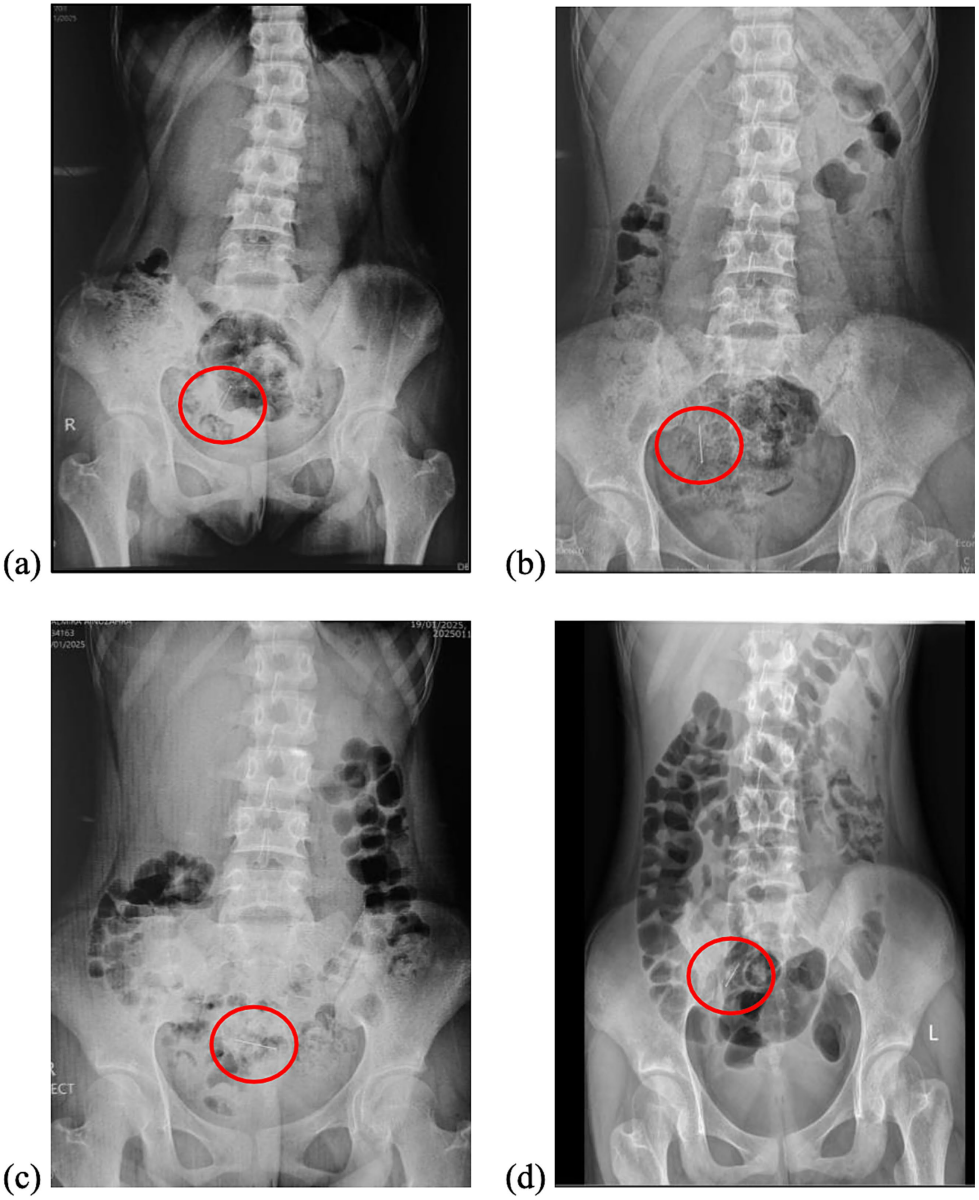
Serial abdominal radiographs demonstrated the presence of a pin (inside the red circle) in the lower quadrant of the abdomen with no acute findings. **(a)** Day of the ingestion (Day 0), **(b)** Day 3, **(c)** Day 7, and **(d)** Day 21.

A 16-year-old female presented with a unique case of a three-week history following the accidental ingestion of a headscarf pin, which occurred while she was adjusting her hijab. Initial evaluation at another medical facility involved conservative management that included the administration of laxatives and serial abdominal X-rays. Repeated imaging consistently revealed the presence of a foreign body, persistently situated in the right lower quadrant of the abdomen (Figure 1).

**Figure 2:**
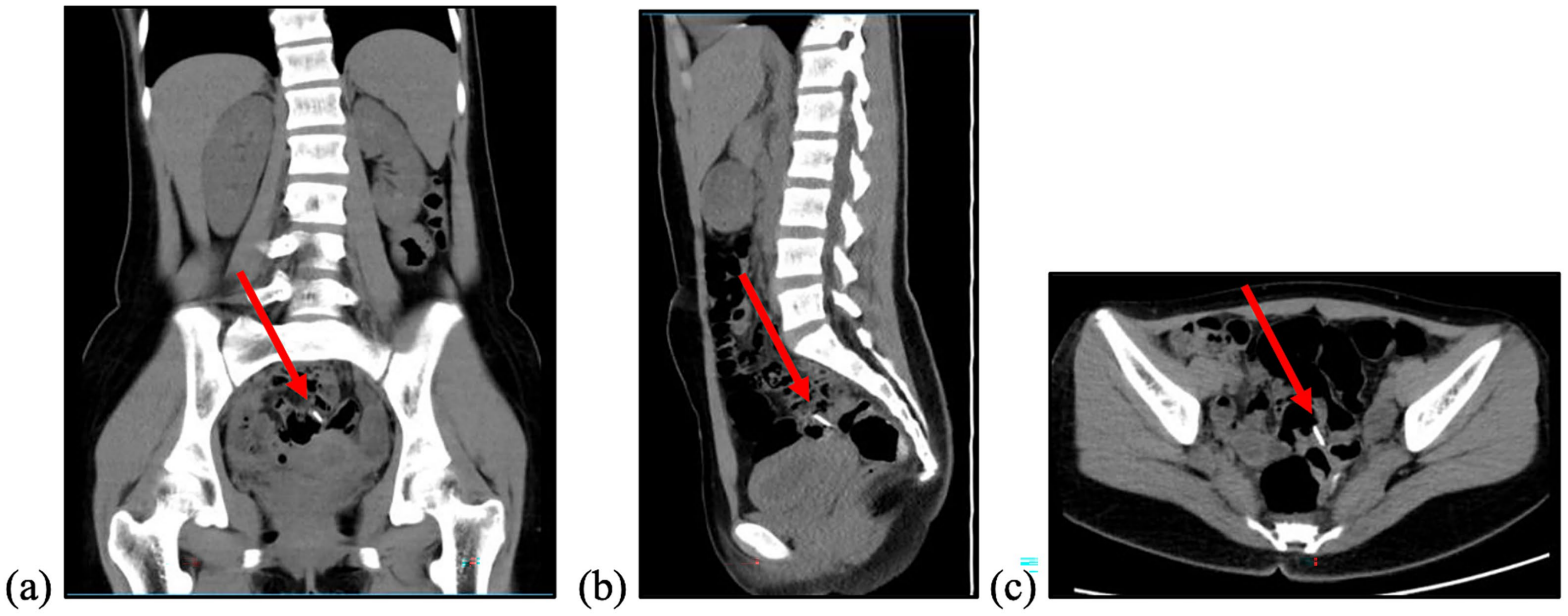
Abdominal CT Scan with contrast performed on Day 23 after ingestion showed a foreign body (red arrow) within the projection of the minor pelvis, within the ileum. **(a)** Coronal view; **(b)** sagittal view; **(c)** axial view.

A colonoscopy conducted at the referring hospital yielded unremarkable findings throughout the lower gastrointestinal tract. Consequently, the patient was referred to our centre for further evaluation. Upon presentation, she reported no symptoms such as abdominal pain, distension, nausea, vomiting, fever, or rectal bleeding, and had no significant past medical history. She was alert, hemodynamically stable, and an abdominal examination revealed normal bowel sounds. Mild tenderness was noted in the right iliac and hypogastric regions without any signs of peritoneal irritation. A repeat endoscopic assessment in our facility revealed several metallic reflections embedded within the intestinal mucosa. Contrast-enhanced abdominal computed tomography (CT) confirmed the absence of gastrointestinal perforation, pinpointing the foreign object in the distal ileum (Figure 2).

The patient was scheduled for laparoscopic exploration. Before trocar insertion, C-arm fluoroscopy was employed to localise the foreign body in real time. The object was clearly identifiable and demonstrated mobility on fluoroscopic imaging. The patient was then placed in the Trendelenburg position, during which the foreign body shifted once more before becoming stationary even when the patient was tilted to the right and left.

**Figure 3:**
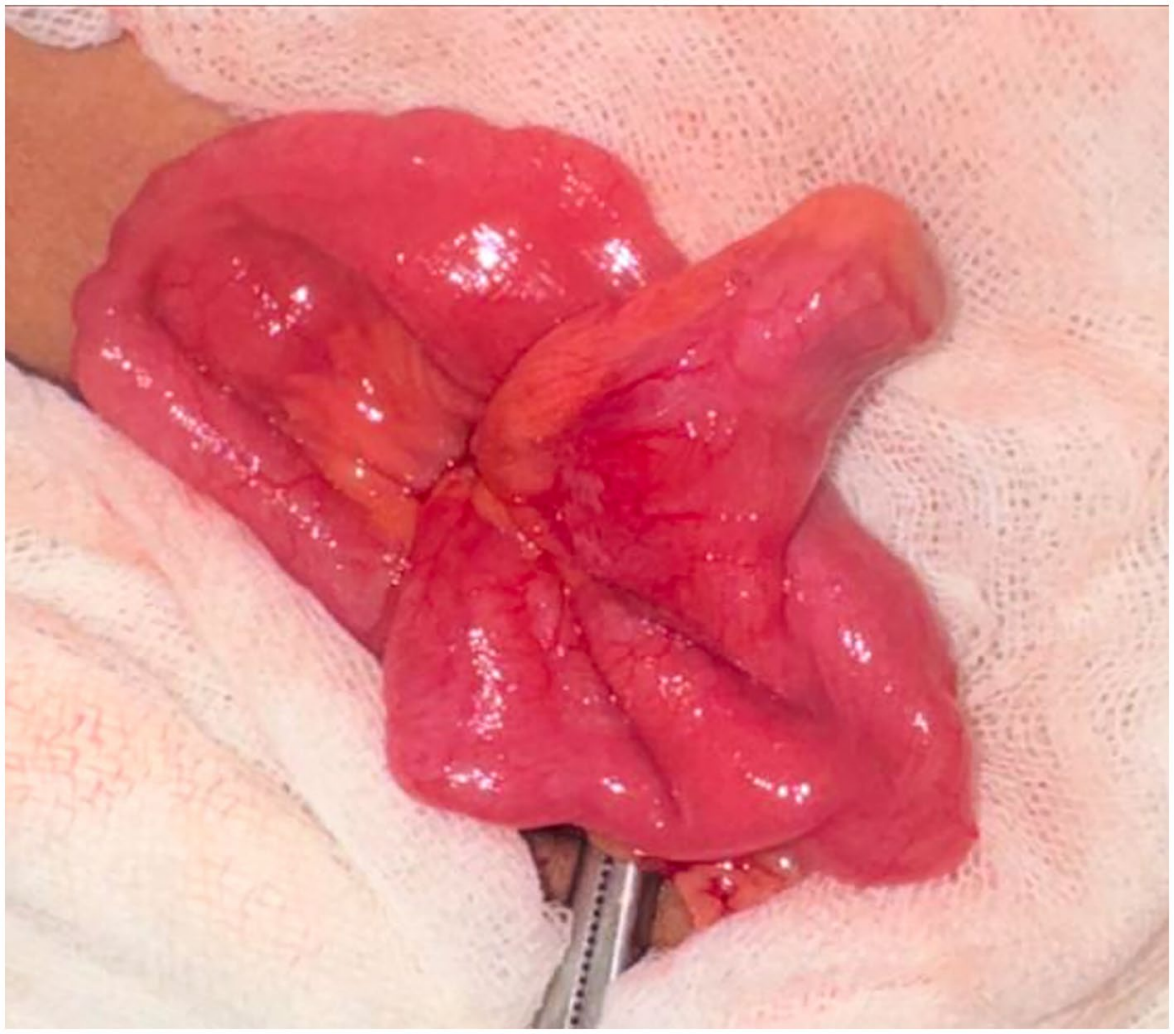
Intraoperative finding.

Laparoscopic exploration was subsequently initiated in this position using three trocars: one infraumbilical and two at the bilateral mid-clavicular lines of the lower abdomen. Exploration was directed toward the patient’s left abdomen, guided by the fluoroscopic findings. While mobilising the small bowel with a grasper, a firm structure was palpated, which corresponded to the foreign body. It was then revealed to be lodged within an incidentally found Meckel’s diverticulum (Figure 3).

A hybrid approach was performed. The diverticulum was exteriorised through the umbilical trocar site, and the foreign body was extracted from its tip. Resection of the Meckel’s diverticulum was subsequently completed using a linear stapler. The headscarf pin was thus successfully retrieved. The postoperative course was uneventful, and the patient was discharged on the third postoperative day.

## Discussion

The accidental ingestion of headscarf pins is a frequent concern among adolescent girls in Muslim-majority regions. This typically occurs because, while adjusting their scarves, these young females often use both hands to wrap the turban around their heads, holding multiple pins, usually four to five, between their teeth as they attach them sequentially. While talking, deep breathing or coughing, these pins can be unintentionally swallowed. A study from India reported that more than half of all patients had ingested headscarf pins, with the highest prevalence among females aged 11 to 20 years (5). Similarly, a cohort study from Israel found that almost three-fourths of cases involved the hijab pin ingestion, with the pin most commonly located in the stomach or duodenum (1).

In most cases, approximately 80 to 90% of foreign bodies that enter the gastrointestinal (GI) tract pass spontaneously within four to six days, including sharp objects like hairpins or needles (5, 6). A 2024 study from a single centre observed that 44.3% of 122 patients who ingested sharp foreign bodies experienced spontaneous passage (7). However, swallowed sharp objects pose significant risks, including gastrointestinal perforation, peritonitis, or, rarely, migration to other organs. A small Indian study involving 75 adolescents who ingested headscarf pins reported a 2.6% incidence of peritonitis (5). While most cases resolve without the need for intervention, impaction can occur, particularly when the object exceeds 6 cm in length, and become lodged at normal anatomical narrowings such as the duodenal loop, pylorus, or ileocecal valve. In rare cases, foreign bodies may become lodged within a Meckel’s diverticulum (6).

This case describes a patient with foreign body ingestion that persisted for three weeks without clear evidence of progression, despite the administration of laxatives, imaging with X-ray and CT scan, and endoscopic evaluation. Since large, sharp objects can sometimes pass through the gastrointestinal tract without causing obstruction, some authors suggest conservative management, despite its potential risks. However, it is crucial to stress the potential risks of this approach to feel the gravity of the situation and the need for proactive measures. A study by Sijabat et al. utilised observation and serial abdominal X-rays to manage a 16-year-old patient who had ingested a needle (6). Similarly, another study by Bezabih and Getu reported the same approach in a 23-year-old male who had swallowed a metal nail (8). In contrast to a study by Li et al., which reported a case of a 27-year-old man who had swallowed a chopstick nine months earlier and presented with dull abdominal pain in the right upper quadrant (RUQ) (9). Other case reports have documented instances where perforation occurred as late as two months after foreign body ingestion (10). The absence of clinical signs and unremarkable plain radiography findings at the initial consultation was insufficient to justify conservative management for foreign body ingestion. The average time from ingestion to perforation was reported to be 10.4 days. If the foreign body has not passed beyond the duodenum, surgical removal is required, even in asymptomatic patients (9).

The ingested pin measured approximately 3 cm in length, whereas the average small bowel lumen diameter in Asian individuals is reported to be 2.75 ± 0.34 cm, according to a cadaveric study from Thailand (11). Given that the pin length exceeded the average luminal diameter, its retention strongly suggested the presence of an anatomical variation providing additional space, such as a Meckel’s diverticulum, which ultimately explained the entrapment site.

Meckel’s diverticulum is a congenital anomaly of the gastrointestinal tract caused by incomplete regression of the vitelline duct. It occurs in approximately 2% of the population (found with equal frequency in both genders), with only 5–6% of those affected developing symptoms. In most cases, the diverticulum of Meckel is accidentally found during laparotomy. This anomaly rarely causes mortality and morbidity (12). To date, the gold standard of identifying Meckel’s diverticulum is through Meckel’s scan, a specific scintigraphy with sensitivity up to 90% and specificity up to 98% in children. However, in adults, the sensitivity is 62.5% with a positive predictive value of 60%. This is because in older human beings, ectopic mucosa, found in Meckel’s diverticulum, detected with scintigraphy may be absent or insufficient (13). Another more widely available modality to identify Meckel’s diverticulum is a CT scan. Without any complication, Meckel’s diverticulum is relatively difficult to identify in the CT scan, even with contrast (14). The structure can be seen as a blind-ended, fluid- or gas-filled formation arising from the small intestine (7). Other than CT, ultrasonography can also help to confirm the existence of Meckel’s diverticulum without the risk of radiation exposure (14, 15). A study conducted by Kawamoto et al. (16) reported that Meckel’s diverticulum was identified more frequently on CT scans in complicated cases (57.1%) compared to asymptomatic cases (42.3%). Overall, CT imaging was able to detect Meckel’s diverticulum in 47.5% of cases. The study also indicates that the amount of peritoneal fat separating the bowel loops can aid in the identification of Meckel’s diverticulum (16).

For the last ten years, several papers have reported patients with sharp foreign body entrapment in Meckel’s diverticulum, which has caused complications. Our case describes a patient who ingested a headscarf pin that had not passed spontaneously even after three weeks. During this period, the patient fortunately exhibited no signs of acute abdomen or peritonitis. Colonoscopy and enteroscopy failed to identify the pin, but a CT scan revealed the foreign body in the distal ileum. Given the fact that the foreign body has not exited the GI tract for more than three weeks, and metallic glints were evident in the distal ileum, the thought of a foreign body in Meckel’s diverticulum should not be excluded. It is also essential to consider that Meckel’s diverticulum may have been missed on CT imaging, especially in the absence of any relevant symptoms, as asymptomatic cases can be radiologically subtle and may be overlooked. A Meckel scan was not done in this case because it is not widely available, especially in developing countries.

In cases where spontaneous passage of a foreign body fails, proceeding with early surgery is a reasonable and safe decision. Laparoscopic exploration is preferred for its minimally invasive advantages and combined diagnostic and therapeutic capability. The addition of intraoperative C-arm fluoroscopy further facilitates more accurate localisation and precise retrieval.

Although intraoperative C-arm fluoroscopy suggested that the foreign body was shifting, it is more likely that the object itself remained lodged while the surrounding small bowel moved. This became evident when the patient was repositioned from neutral to Trendelenburg. The apparent displacement of the object mirrored the change in body position, confirming its location within a mobile hollow viscus, namely the small bowel. At that point, the object shifted once more before becoming stationary. Once a foreign body is observed to remain fixed under fluoroscopy, minimally invasive exploration can be safely initiated, with dissection focused on the abdominal quadrant indicated by the fluoroscopic findings.

## Conclusion

Meckel’s diverticulum, though rare, should be suspected in cases of prolonged foreign body retention. Standard imaging may fail to identify this anomaly, particularly in asymptomatic patients. Early laparoscopy provides both diagnostic and therapeutic advantages in such scenarios, and persistent foreign body retention should always raise suspicion for Meckel’s diverticulum, where minimally invasive exploration ensures safe and definitive management. In line with current literature, early exploration with a minimally invasive approach is warranted when spontaneous passage fails within 10 days, especially for sharp or potentially hazardous objects.

## Data Availability

The raw data supporting the conclusions of this article will be made available by the authors, without undue reservation.
