# Supplementary material for: Tackle the Meckel: laparoscopic diverticulectomy for a retained ingested foreign body—a case report
Source: Front Med (Lausanne). 2025 Oct 1;12:1650643. doi: 10.3389/fmed.2025.1650643 (PMC12521437; doi:10.3389/fmed.2025.1650643)
Supplement: Supplementary file 1 [file Supplementary_file_1.docx]

**Supplementary Material**

Pin ingested

Repeated Abdominal

X-Ray

Colonoscopy

Last Hospital

CT Scan

Endoscopy

Surgery

Previous Hospital

Persistent foreign

body

No abnormality

Embedded metallic reflection

Foreign body (+)

18 Days

3 Days

Discharge

3 Days

**Supplementary 1.** Chronological summary of the patient’s management

Patient in Lloyd-Davies + Trendelenburg

C-Arm

Pin in pelvic minor

Patient in Lloyd-Davies + neutral

Pin in pelvic minor, shift to the left of vertebrae

Insert trocar infraumbilically + lower abdomen in bilateral mid-clavicular line

Focusing on the left lower abdomen

Start laparoscopy exploration

Finding of a foreign body within Meckel’s

Hybrid mini-incision

Exteriorize Meckel’s diverticulum

Retrieve pin from the tip

Excise the diverticulum (linear stapler)

Finishing surgery

**Supplementary 2.** Workflow of laparoscopic and hybrid management of a foreign body within Meckel’s diverticulum
